# Stochastic epigenetic mutations (DNA methylation) increase exponentially in human aging and correlate with X chromosome inactivation skewing in females

**DOI:** 10.18632/aging.100792

**Published:** 2015-08-23

**Authors:** Davide Gentilini, Paolo Garagnani, Serena Pisoni, Maria Giulia Bacalini, Luciano Calzari, Daniela Mari, Giovanni Vitale, Claudio Franceschi, Anna Maria Di Blasio

**Affiliations:** ^1^ Istituto Auxologico Italiano IRCCS, Cusano Milanino, 20095 Milan, Italy; ^2^ Department of Experimental, Diagnostic and Specialty Medicine, Alma Mater Studiorum- University of Bologna, Bologna 40138, Italy; ^3^ Interdepartmental Center “L. Galvani”, University of Bologna, Bologna 40126, Italy; ^4^ Geriatric Unit, IRCCS Ca' Granda Foundation Maggiore Policlinico Hospital, Milan, Italy; ^5^ Department of Clinical Sciences and Community Health, University of Milan, Milan, Italy

**Keywords:** epimutations, DNA methylation, aging, X chromosome inactivation skewing, epigenetics

## Abstract

In this study we applied a new analytical strategy to investigate the relations between stochastic epigenetic mutations (SEMs) and aging. We analysed methylation levels through the Infinium HumanMethylation27 and HumanMethylation450 BeadChips in a population of 178 subjects ranging from 3 to 106 years. For each CpG probe, epimutated subjects were identified as the extreme outliers with methylation level exceeding three times interquartile ranges the first quartile (Q1-(3 × IQR)) or the third quartile (Q3+(3 × IQR)). We demonstrated that the number of SEMs was low in childhood and increased exponentially during aging. Using the HUMARA method, skewing of X chromosome inactivation (XCI) was evaluated in heterozygotes women. Multivariate analysis indicated a significant correlation between log(SEMs) and degree of XCI skewing after adjustment for age (β = 0.41; confidence interval: 0.14, 0.68; p-value = 0.0053). The PATH analysis tested the complete model containing the variables: skewing of XCI, age, log(SEMs) and overall CpG methylation. After adjusting for the number of epimutations we failed to confirm the well reported correlation between skewing of XCI and aging. This evidence might suggest that the known correlation between XCI skewing and aging could not be a direct association but mediated by the number of SEMs.

## INTRODUCTION

In multicellular organisms, specificity of cell types is maintained by mitotically heritable differences in gene expression, which are in part regulated by epigenetic mechanisms. These include RNA-based mechanisms, histone modifications, and DNA methylation [1]. The full range of epigenetic marks is currently unknown but is potentially enormous, considering that the diploid human epigenome contains >10^8^ Cytosines (of which >10^7^ are CpGs) and >10^8^ histone tails that can all potentially vary.

DNA methylation is one of the best understood epigenetic modification and has an important role in several biological processes such as genome imprinting, defence against viral sequences, inhibition of recombination, as well as assembly of heterochromatin [2].

Aberrant DNA methylation patterns have been linked to genomic instability and increased mutation rates [3,4]. The role of DNA methylation has been mainly explored in the context of cancer [5,6]. Findings from these studies have extensively demonstrated that cancer development is associated with gain of DNA methylation at CpG islands, loss-of-imprinting and epigenetic remodeling of repeat elements [7]. Interestingly, altered DNA methylation seems to be involved in the pathogenesis of other age-related diseases, such as cardiovascular, neurological and metabolic disorders, and autoimmune diseases [8].

Recently published papers also demonstrate that DNA methylation patterns are not static and they naturally change with aging in a complex manner. The biological meaning of these changes remain to be elucidated although many authors suggest that aging-associated epigenetic modifications may play a central role in the development of several age-related diseases [9,10].

Several studies analysed the DNA methylation status in groups of subjects going from childhood to centenarian age through a genome-wide approach and reported an age-related decrease in global DNA methylation [11–13]. Important insights have also been gained regarding the genes showing promoter hyper- or hypomethylation as function of age [13–18].

Age-associated hypermethylation preferentially affects loci at CpG islands and genes involved in developmental functions and in the control of metabolism [13,19]. Conversely, hypomethylation seems to mainly involve repetitive elements like Alu sequences [13].

Technologies able to investigate the epigenetic profile have recently reached the stage at which large-scale studies are becoming feasible. Next Generation Sequencing can be considered the future challenge, although array-based methods actually represent the most suitable and cheap devices for genome wide epigenetic studies. The increasing interest in epigenome-wide association studies (EWASs) has supported the development of a growing number of analytical tools and packages for the analysis of array methylation data [14,20–25].

Usually, after quality control step and normalisation of the dataset, mean methylation levels are calculated for each CpG site and are compared between groups of subjects in order to identify significant differences or correlations. This approach is useful and powerful to identify epigenetic alterations shared by a group of subjects and potentially associated with their phenotype. It provides a general overview of the effect size but does not reflect differences in variances or other features of the methylation spectrum.

Rare or stochastic epimutations that are not shared among subjects and that minimally affect the mean methylation level of the group remain unexplored, although they may play a role in phenotype development. Furthermore, an analytical strategy based on comparisons of mean methylation values does not allow to process data obtained from single subjects.

As an example, studies on aging well depicted common epigenetic modifications associated to the aging process, however there is still a lack of knowledge regarding the rate of epigenetic mutations that stochastically arise on the genome and that are not shared among subjects.

To investigate this issue we propose herein a different analytical approach that allows to identify stochastic epigenetic mutation (SEMs) not shared among subjects. We applied this analytical strategy to investigate the relations between stochastic epigenetic alteration and aging. Furthermore, we studied the relation between SEMs and age-dependent skewing of X chromosome inactivation that represents a phenomenon favoring the expression of multiple deleterious alleles and consequently influencing human health [26].

## RESULTS

### Overall DNA methylation is correlated with age and BMI

Methylation levels of 25.014 CpG sites were evaluated in whole blood from 170 subjects ranging from 3 to 106 years. A first descriptive evaluation of the methylation status was performed using the Principal Component Analysis (Supplemental Figure 1). This analysis excluded the presence of samples with aberrant epigenetic profiles and the presence of clusters of samples carrying peculiar epigenetic features. Subjects were then grouped in 5 age ranges (0-19 years, 20-39 years, 40-59 years, 60-79 years, 80-106 years) including at least 10 subjects in each age range.

**Figure 1:**
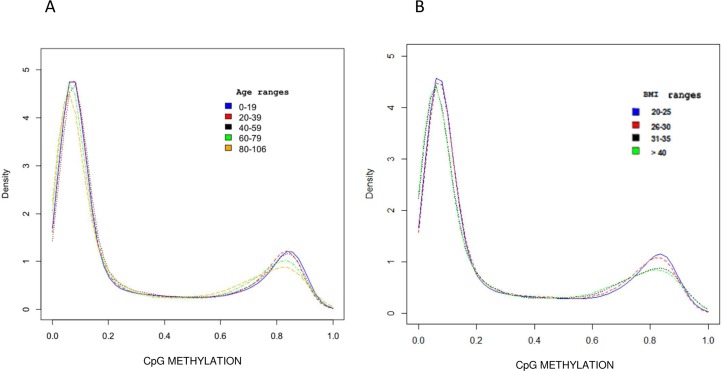
DNA Methylation profile distribution The density plot describes the mean methylation profile of samples grouped by age range **(A)** and by BMI range **(B)**.

As shown in Figure 1A, global DNA methylation β levels progressively decreased with age and were inversely correlated with aging (β = −0.36; confidence interval = −0.48, −0.22; p-value = 1.43 × 10^−6^). Moreover, a significant progressive decrease in overall DNA methylation levels was also associated with BMI, as shown by the density plot in Figure 1B. Indeed, DNA methylation was inversely correlated with BMI also after adjustment for age (β = −0.19642; confidence interval: −0.37, −0.02; p-value < 0.05).

### The number of epimutations increases exponentially during aging

**Figure 2:**
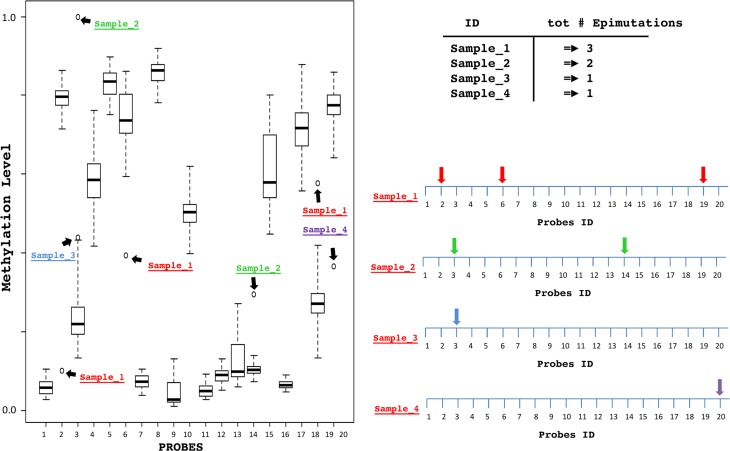
Schematic description of the SEM For each probe, a Box-and-whiskers plot analysis identifies extreme outliers samples. For each sample epimutations are detected, counted and mapped considering their genomic position.

The distribution and variability of methylation levels were studied for each one of the 25.014 CpG sites using Box-and-whiskers plots (Figure 2) as described in the materials and methods section. This allowed to obtain the total number of SEMs for each subject.

**Figure 3:**
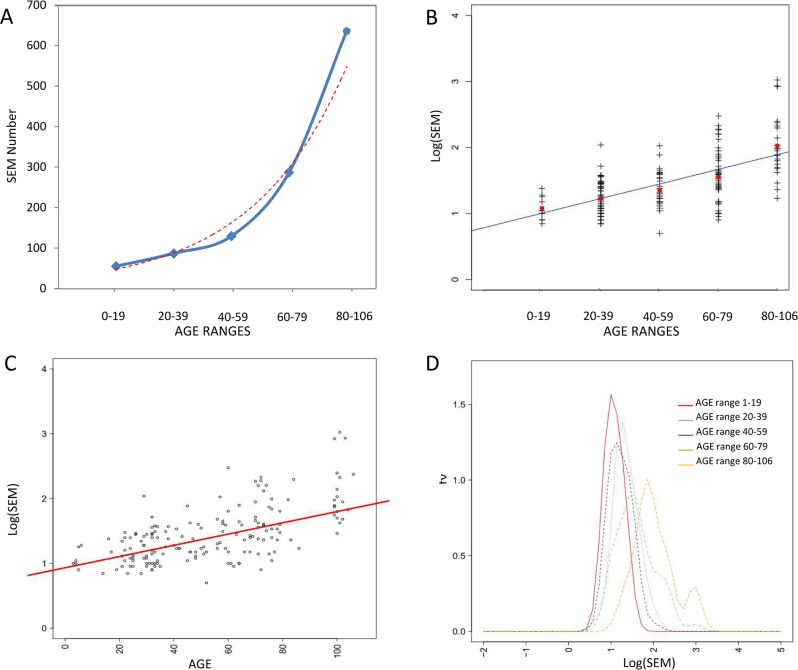
Correlations between epimutations and aging **(A)** Exponential relation between age ranges and number of SEM. **(B)** Linear correlation between age ranges and log(SEM), red squares indicates mean log(SEM) value. **(C)** Linear correlation between age log(SEM) considering all samples independently. **(D)** Density plot describing age dependent shift in log(SEM).

As shown in Figure 3A, considering age ranges, the mean number of SEMs increased exponentially during aging.

SEMs data were not normally distributed and thus were logarithmic transformed before regression analysis. A strong correlation between age ranges and log(SEM) was evident (η= 0.92) (Figure 3B). This correlation was still significant when the subjects were not grouped in age ranges but considered individually (r = 0.63; confidence interval: 0.53, 0.71; p-value = 2.2 × 10^−16^)(Figure 3C).

The density plot in Figure 3D shows the age dependent shift in SEMs frequency depending on age range.

Multivariate analysis performed considering log(SEM), overall DNA methylation levels, BMI and aging confirmed that these findings were not influenced by the overall DNA methylation levels. Interestingly, no significant correlation between log(SEMs) and BMI even after adjustment for age and other covariates was detected.

### The number of SEMs is correlated with skewing of X chromosome inactivation

Using the HUMARA method, skewing of X chromosome inactivation (XCI) was evaluated in a subset of 45 heterozygous women. Multivariate analysis indicated a significant correlation between log(SEM) and degree of XCI after adjustment for age (β = 0.41; confidence interval: 0.14, 0.68; p-value = 0.0053).

**Figure 4:**
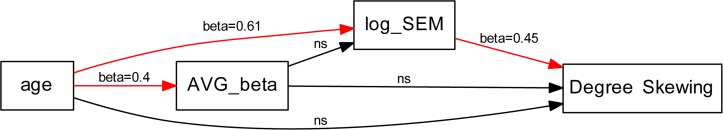
Path diagram with associated path coefficients (β) Arrows indicate the interrelationships tested in the analysis: red arrows indicate significant relationships (p value < 0.05), black arrows indicate non-significant relationships AVG beta = average DNA methylation β values.

Subsequently, we performed a PATH analysis using the not imputed original dataset to test the complete model containing the variables skewing of XCI, age, log(SEMs) and overall CpG methylation in order to understand the relations between them. After adjusting for the number of epimutations, the marginal correlation between skewing of XCI and aging was not significant yet. Figure 4 shows the path with the respective standardized coefficients. Similar results were obtained also using and combining the five imputed datasets.

## DISCUSSION

Recent findings demonstrate that age associated epigenetic modifications involve specific array of genes that become hyper or hypo-methylated [11–16].

In addition to this common epigenetic drift that involves specific loci and is shared among old subjects, there are also stochastic epigenetic mutations that arise randomly in the genome and that are not shared among subjects.

The somatic mutation theory of aging claims that accumulation of stochastic mutations in somatic cells results in a decrease of cellular functions [27] and plays an important role in aging and in several age-related diseases. Ong et al. recently reported a massive increase in variably methylated region with age and in regions of the genome associated with open chromatin and neurotransmission [21]. However, to date there are no data regarding the accumulation rate of epigenetic mutations.

In the present study we investigated this specific topic with a new analytical strategy. Briefly, in a population of normal subjects we estimated, for each locus, the normal methylation range. This allowed us to identify epimutations when the methylation value of a subject exceeded the normal range and was extremely far from that of the other subjects. We validated this type of analysis using duplicated samples and samples with well-known and previously reported epigenetic alteration.

Using this analytical approach, we report for the first time that the number of SEMs is low in childhood and increases exponentially during aging. It is highly variable among individuals but the correlation between log(SEMs) and age is extremely strong. These findings lead to speculate that SEMs might have a predictive value for aging and could be used as an index of the biological age.

We also confirmed that overall DNA methylation level decreases during aging and observed that, independently of age, BMI can influence DNA methylation. These data are in line with previous studies reporting an inverse correlation between BMI and LINE-1 methylation [28]. In contrast, no association between BMI and the number of SEMs was detected. This observation is worth noting and unexpected if considering the association between BMI and food intake that, by itself, can deeply influence DNA methylation [29–31]. Finally, we investigated the relationship between the number of SEMs and XCI skewing. This phenomenon is considered a natural consequence of aging [32] and it has been reported to be associated with several age-related diseases [33–38]. Moreover, recent findings suggest that XCI skewing might also influence human longevity and life span [26].

We have described a significant correlation between XCI skewing and the number of observed SEM. Interestingly, the correlation between age and XCI skewing, previously reported in other studies [32,39,40] did not remain significant when the number of SEMs was considered as a covariate. These results may suggest for the first time that XCI skewing may not be a direct consequence of aging. Indeed, this phenomenon has been recently widely debated. Some studies have proposed that it could be the result of a clonal stochastic loss of haematopoietic cells [36,41] or of a competitive advantage for haematopoietic stem cells with a specific genotype [42]. The data presented herein support this hypothesis leading to speculate that an increased number of SEMs might influence haematopoietic stem cells viability or might create conditions able to induce clonal stochastic loss of a specific type of haematopoietic cells.

We acknowledge that the present study has few limitations:

1. Only DNA from blood cells was analyzed and, thus, additional studies on other tissues are mandatory to confirm these preliminary observations.
2. The phenotypic data of the subjects studied are rather scanty. Future studies based on a detailed phenotypic characterization may better investigate the effect of SEMs on human health and confirm their role as a predictor of human aging and healthy aging.
3. The number of subjects enrolled is consistent, but we must underline that a larger sample size could better estimate normal ranges of DNA methylation for each locus.

In conclusion, the analytical approach presented herein might be useful to identify for each subject of a population the pattern of epimutations that could have a role in determining his phenotype. Moreover, we report for the first time that the number of SEMs increase exponentially during aging. This observation might be used as a predictor of aging and might have important implications in future. Finally, we present evidence suggesting that the known association between XCI skewing and aging could be mediated by the number of stochastic epimutations.

## METHODS

### Study population

We enrolled a total number of 178 subjects spanning from 3 to 106 years selected from Istituto Auxologico Italiano Biobank. In order to better estimate epimutations frequency we identified 5 age ranges (0-19 years, 20-39 years, 40-59 years, 60-79 years, 80-106 years), enrolling at least 10 subjects for each age range. An histogram of age and BMI distribution of subjects is shown in Supplemental Figure 2.

Subjects with imprinting disorders, emathological cancers, immune diseases were excluded from the study. The study protocol was approved by the Ethical Committee of the Istituto Auxologico Italiano.

### DNA extraction and bisulphite treatment of the DNA

Genomic DNA was extracted from peripheral blood using the Wizard genomic DNA purification kit (PROMEGA, Madison WI, USA). Samples were processed for DNA isolation as follows: 3 to 5 mL of blood were lysed with lysis solution provided, digested with proteinase K in sodium dodecyl sulfate (SDS) buffer at 37°C for 1 hours, then DNA was extracted by salting-out, and resuspended in Tris-EDTA (TE) buffer. Sodium bisulphite conversion of DNA (500 ng) was performed by the EZ DNA Methylation-Gold Kit (Zymo Research Corporation, Orange, CA) according to the manufacturer's protocol.

Quality control and quantification of DNA were performed before and after bisulphite conversion. DNA was quantified with NanoDrop (NanoDrop Products Thermo Scientific Wilmington, DE) and quality was assessed by visualisation of genomic DNA on 1% agarose gel electrophoresis. Only DNA samples not fragmented were subsequently processed.

### Genome-wide methylation analyses

Whole-genome methylation analysis of the DNA from 178 subjects was performed using the Illumina Infinium Methylation Platform (Illumina, San Diego, CA). 94 samples were analysed using the Infinium HumanMethylation 450K BeadChip while 84 samples were analysed using the 27K BeadChip. Those chips allow to assess the methylation level of 485,764 and 27,578 CpG sites over the entire genome, respectively.

The methylation profile was analysed according to the manufacturer's instructions using Illumina-supplied reagents and conditions. In brief, after bisulfite conversion, 250 ng of DNA were whole-genome amplified (WGA) and enzymatically fragmented. The bisulfite-converted WGA-DNA samples were purified and randomized again prior to hybridization to BeadChips. During hybridization, the WGA-DNA molecules annealed to locus-specific DNA oligomers linked to individual bead types, one designed against the unmethylated site and one against the methylated site. After hybridization, allele-specific single-base extension provided another level of specificity and incorporated a fluorescent label for detection. The level of methylation was determined at each locus by the intensity of the two possible fluorescent signals, specific for the methylated and unmethylated alleles. DNA methylation values, described as β-values, were recorded for each locus in each sample. Beta-value provides a continuous measure of levels of DNA methylation at a CpG site, ranging from 0 in the case of completely unmethylated sites to 1 in completely methylated sites.

### HUMARA assay

X-inactivation pattern was determined in 45 female blood DNA samples using a modification of the methylation analysis of the HUMARA locus as described previously (26-GENTILINI). Briefly, 250 ng of DNA were digested at 37°C for 2 h with 10 U HpaII and 10 U HhaI (CELBIO) and a no-enzyme control digest was also setup for each sample. Digested and undigested DNAs were then amplified in duplicate PCRs using primers, amplifying the highly polymorphic CAG repeat region in Exon 1 of the AR gene at Xq12. The sequences of the primers used were as follows: forward; 5′-GCT GTG AAG GTT GCT GTT CCT CAT-3′ labelled with 5′-phosphoamidite dye, reverse; 5′-TCC AGA ATC TGT TCC AGA GCG TGC-3′. The samples were amplified for 35 cycles comprising of 15 s at 95°C, 30 s at 62°C and 30 s at 72°C with an initial denaturation at 95°C for 5 min. The PCR products were separated on an ABI 310 automated sequencer. The size of PCR product from each allele was analysed by Genescan software for the quantification by peak height.

XCI skewing was measured as Degree of Skewing (DS), which designates the percentage of the preferentially active allele. DS varies between 0% and 50%, where 0% indicates a random X inactivation pattern and 50% a completely skewed inactivation pattern.

### Methylation data management, normalisation and quality control

Methylation raw data were generated using GenomeStudio software (Illumina, San Diego, CA)and were pre-processed using IMA R package [23]. Samples with low bisulphite conversion (BS) efficiency (BS control intensity values < 4000) were immediately excluded from the analysis as well as samples that failed the quality control analysis performed according the IMA package pipeline. Moreover, a principal component analysis was performed on methylation data in order to identify and remove samples with aberrant methylation profiles. A total of 8 samples were excluded from the study. Density plot of β-values showed a bimodal distribution with a shift between the positions of the peaks derived from type I and type II assay. Therefore, peak correction was performed using the IMA package, moreover data were normalised using background correction followed by quantile normalisation method. Furthermore, probes containing missing β-values, probes having <95% of samples with detection p-value <0.05 and probes on chromosome X and Y were also removed from the analysis.

We finally pooled data obtained from 27K and from 450K arrays and created a new data matrix containing 25.014 probes that were in common between the two array platforms and passed quality control steps.

### Epimutations detection

The distribution and variability of methylation levels in our population were studied for each one of the 25.014 CpG sites using Box-and-whiskers plots in order to identify SEMs. For each probe, whenever the methylation level of one subject extremely differed from the rest of the population we considered the outlier sample as epimutated for that locus.

Thus, for each locus epimutated subjects were identified as the extreme outliers with methylation level exceeding three times interquartile ranges Q1-(3 × IQR) and Q3+(3 × IQR). Finally, all epimutated loci were annotated in a new data matrix that allowed to calculate, for each subject, the total amount of epimutations and their genomic position.

A schematic description of the analysis is shown in Figure 2.

The Box-and-whiskers plot analysis was conducted using boxplot function provided in the R *car* package and confirmed using the outlier function in the R *outliers* package.

### Validation of the SEM analysis

In order to confirm the power of this analytical approach to detect epimutations we performed two separate tests after introducing positive controls.

1. We analysed 3 samples in duplicates and compared epimutations found in each of them. Results showed a mean correlation of 0.99 p< 0.01 among the experiments. It's important to underlie that the duplicate samples underwent independent bisulfite conversion reactions and this suggests that epimutations are not significantly influenced by bisulfite conversion errors.
2. We analysed 48 whole blood DNA samples obtained from subjects affected by imprinting diseases (BWS syndrome, Angelman Syndrome and Silver Russel syndrome) that attended diagnostic test at Istituto Auxologico Italiano. For these subjects we already had a medical report indicating the genomic position of their epigenetic alteration. For this control test, we used only data obtained from Illumina Infinium 450K arrays thus reducing the number of the control population (n=91). However, using a more informative array, we were able to better define all probes showing epigenetic alterations. Briefly, after the identification of the epimutated probes we performed a test for overrepresentation of epimutated probes inside each gene using the hypergeometric cumulative function. We considered the total number of probes (N_G_ = 485.764), the total number of epimutated probes in the selected sample (N_S_) the number of probes in each gene (G_I_) and the epimutated probes in each gene (S_I_).

The analysis identified genes with enriched number of epimutated probes (bonferroni corrected p-value < 0.05) confirming the presence of the epigenetic alterations previously reported in the medical report.

Supplemental Figure 3 shows positive control test results.

All the R scripts employed as well as the set of methylation data generated will be made freely available to the scientific community.

### Multiple regression and path analysis

The variables (age, BMI, overall methylation level, Skewing of XCI and log(SEM))were Z transformed and Z scores were used for the multiple regression and path analysis. Results of regression analyses were indicated as standardised regression coefficients β.

The univariate and multivariate linear regressions were conducted using the generalised linear mixed model function provided in the R base package.

The interrelationships among skewing of XCI and the other variables were examined by a path analysis model. A path diagram with associated path coefficients (β) was constructed as shown in Figure 4 based on previous findings [13] and theoretic rationales.

As age is generally considered a determinant of changes in XCI skewing profile and in overall DNA methylation, direct and indirect Paths from age to these components were tested. The Path Analysis was performed using the *General Structural Equation Models* R package.

### Missing data imputation

Multiple imputation, producing five imputed datasets, was carried out to allow full use of all available data.

Imputation was performed using the bootstrap-based EMB algorithm included in the R package Amelia II. Five imputations were generated and models fitted to each imputed dataset. Model results were consolidated using Rubin's rules [43].

## SUPPLEMENTARY MATERIAL FIGURES


