# Supplementary material for: Stochastic epigenetic mutations (DNA methylation) increase exponentially in human aging and correlate with X chromosome inactivation skewing in females
Source: Aging (Albany NY). 2015 Aug 23;7(8):568–76. doi: 10.18632/aging.100792 (PMC4586102; doi:10.18632/aging.100792)
Supplement: Supplementary file 1 [file aging-07-568-s001.pdf]

SUPPLEMENTAL FIGURES

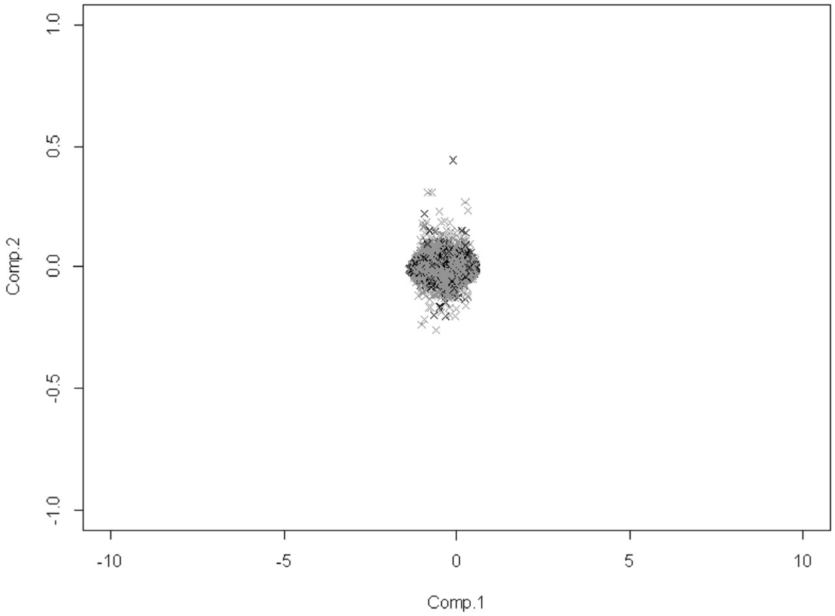

Supplemental Figure 1. Principal component Analysis of Methylation Values indicates that methylation profile of samples analysed is very similar among subjects

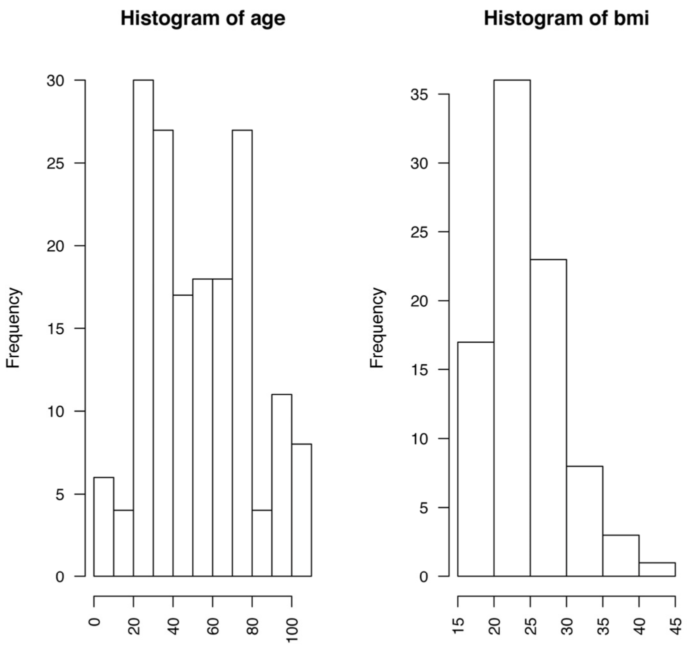

Supplemental Figure 2. Histogram describing age and bmi distribution of the population enrolled.

EXAMPLE: Beckwith-Wiedemann Syndrome

A.1

A.2

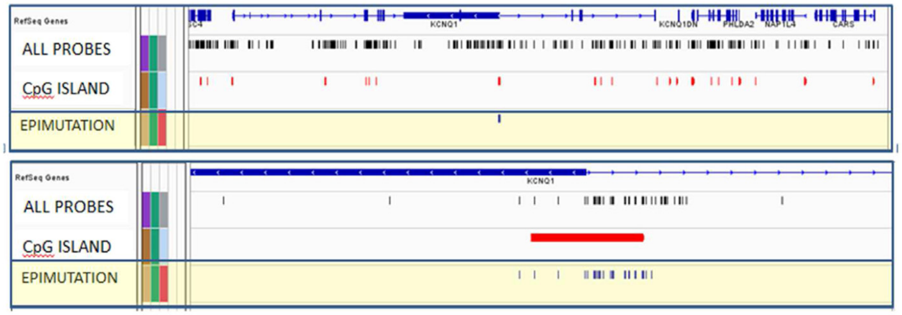

EXAMPLE: Silver Russell Syndrome

B.1

B.2

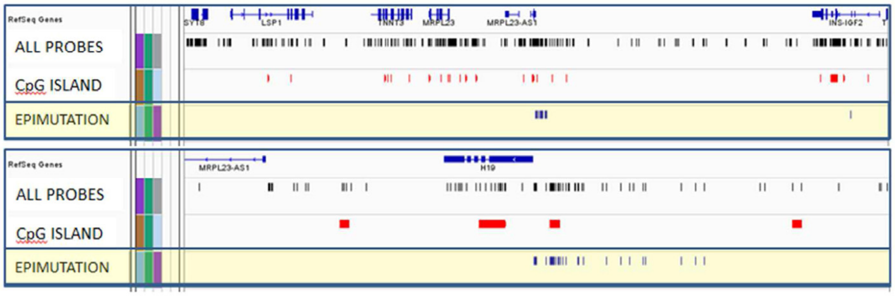

Supplemental Figure 3. Validation of the analytical approach. The picture shows a graphic representation of probes resulted epimutated in samples carrying a previously described epigenetic defect. **(A.1)** The Figure describes a case of Beckwith-Wiedemann Syndrome. In black the cohordinates of all probes, in red the position of CpG island and in blue the genomic position of probes resulted epimutated. **(A.2)** Magnification of the region with the epigenetic alteration. **(B.1)** The Figure describes a case of Silver Russel Syndrome. In black the cohordinates of all probes, in red the position of CpG island and in blue the genomic position of probes resulted epimutated. **(B.2)** Magnification of the region with the epigenetic alteration.
